# Detection of H5N1 HPAIV Clade 2.3.4.4b Avian Influenza Virus in Backyard Chickens in Costa Rica

**DOI:** 10.3390/v18070799

**Published:** 2026-07-20

**Authors:** Bernal León, Hebleen Brenes, Nidia S. Trovao, Olga Aguilar, Fabián Carvajal, Idania Chacón, Guisella Chaves, Mónica Guzmán, Claudio Soto-Garita, Estela Cordero, Francisco Duarte-Martínez, Trushar Jeevan, Richard Webby, Adam Rubrum, Randall Arguedas, Ronaldo Chaves

**Affiliations:** 1 Laboratorio LSE-LANASEVE, Servicio Nacional de Salud Animal (SENASA), Heredia 40104, Costa Rica; oaguilara@senasa.go.cr (O.A.); fcarvajal@senasa.go.cr (F.C.); ichacon@senasa.go.cr (I.C.); gchavesg@senasa.go.cr (G.C.); mguzman@senasa.go.cr (M.G.); 2 Centro Nacional de Referencia en Virología (CNRV), Instituto Costarricense de Investigación y Enseñanza en Nutrición y Salud (Inciensa), Tres Ríos, Cartago 30301, Costa Rica; hbrenes@inciensa.sa.cr (H.B.); csoto@inciensa.sa.cr (C.S.-G.); 3 Department of Pathobiology, College of Veterinary Medicine, University of Illinois Urbana-Champaign, Urbana, IL 61802, USA; ntrovao@illinois.edu; 4 Carl R. Woese Institute for Genomic Biology, University of Illinois Urbana-Champaign, Urbana, IL 61802, USA; 5 National Center for Supercomputing Applications, University of Illinois Urbana-Champaign, Urbana, IL 61802, USA; 6 Laboratorio de Genómica y Biología Molecular, Centro Nacional de Referencia en Inocuidad Microbiológica de Alimentos (CNRIMA), Instituto Costarricense de Investigación y Enseñanza en Nutrición y Salud (Inciensa), Tres Ríos, Cartago 30301, Costa Rica; ecordero@inciensa.sa.cr (E.C.); fduarte@inciensa.sa (F.D.-M.); 7 Department of Host-Microbe Interactions, St. Jude Children’s Research Hospital, Memphis, TN 38105, USA; trushar.jeevan@stjude.org (T.J.); richard.webby@stjude.org (R.W.); adam.rubrum@stjude.org (A.R.); 8 DRM-Di-Ochomogo Costa Rica, Servicio Nacional de Salud Animal (SENASA), San Nicolas, Cartago 30104, Costa Rica; rarguedasp@senasa.go.cr; 9 Programa Nacional de Salud Aviar, Departamento de Epidemiología, Servicio Nacional de Salud Animal (SENASA), Ulloa, Heredia 40104, Costa Rica; rchavesl@senasa.go.cr

**Keywords:** HPAIV, backyard chickens, clade 2.3.4.4b, Costa Rica

## Abstract

Influenza A virus is a segmented, negative-sense RNA virus. Since the early 2020s, H5 clade 2.3.4.4b viruses have spread widely across Europe, Africa, and Asia, affecting wild birds and poultry. Costa Rica reported its first H5 clade 2.3.4.4b avian influenza virus (AIV) case on 19 January 2023. This study describes an outbreak in backyard chickens and ducks. Initial serum samples collected on 24 January showed three chickens negative for AIV, while one duck tested positive by ELISA and agar gel immunodiffusion (AGID). During a second visit on 27 January, three of four chicken sera collected tested positive by ELISA and AGID. Tissue samples were positive for influenza A by qRT-PCR. Next-generation sequencing recovered five of the eight viral genomic segments, and the hemagglutinin cleavage site sequence (REKRRKR↓G) confirmed a highly pathogenic avian influenza virus (HPAIV) H5 strain. The samples were submitted to the National Veterinary Services Laboratories for confirmation. Serological testing showed reactivity to North American low pathogenic H5 antigens, and qRT-PCR amplified influenza A and N1 genes. Virus isolation and next-generation sequencing (NGS) of all eight viral genome segments were successfully performed at the WHO Collaborating Centre at St. Jude Children’s Research Hospital (SJCRH).

## 1. Introduction

Influenza A is a segmented negative-sense RNA virus that continually evolves through antigenic drift and antigenic shift during replication in natural reservoir host species where it diversified into multiple subtypes. Since early 2020, influenza viruses of the H5 subtype carrying an HA gene of GS/GD/96 clade 2.3.4.4b have expanded their geographical and host ranges leading to the loss of millions of domestic birds in Europe, Africa, and Asia [1]. Conversely, waterfowl of the subfamily *Anatinae* can carry this virus without showing clinical signs, enhancing virus spread. Infections with the highly pathogenic H5N1 clade 2.3.4.4b avian influenza virus have been detected in chickens and other wild birds in North America since at least December of 2021 [2]. In October 2022, the H5N1 virus was officially reported in Colombia; it was later reported in Peru, Ecuador, and Venezuela, signaling a southward spread [3]. 

In November 2022, Peruvian pelicans (*Pelecanus thagus*) along the coast and on offshore islands of Peru experienced a mass die-off [4]. During the same month, an H5N1 outbreak was declared in poultry in Ecuador, affecting more than 1.1 million poultry on two farms by February 2023 [5].

In early December 2022, increased numbers of wild bird deaths were detected across the north coast of Chile and H5N1 viruses were detected in 93 samples. Phylogenetic analysis of sequences from nine of these samples suggested multiple viral introductions into South America [3].

In Uruguay, the index outbreak of H5N1 was detected on February 14, 2023, in Laguna Garzón, where two black-necked swans (*Cygnus melancoryphus*) were found dead. All backyard poultry subsequently infected in Uruguay resided near ponds or small lagoons, where they often interacted with waterfowl, gulls, and shorebirds. This aligns with the scenario in which highly pathogenic avian influenza virus (HPAIV) is introduced into poultry and backyard populations through the activity of wild aquatic birds [6]. This spread can be mediated through indirect contact; waterfowl may transmit the virus to an area where it can then spread to poultry through other contaminated material [6].

In Costa Rica, the first case of H5N1 was identified on 19 January 2023, in a Pelican *Pelecanus occidentalis* found on the southern Atlantic coast [7]. The objective of this study is to provide a detailed description of the unique and particular case involving backyard chickens in Costa Rica in 2023.

## 2. Materials and Methods

**Figure 1:**
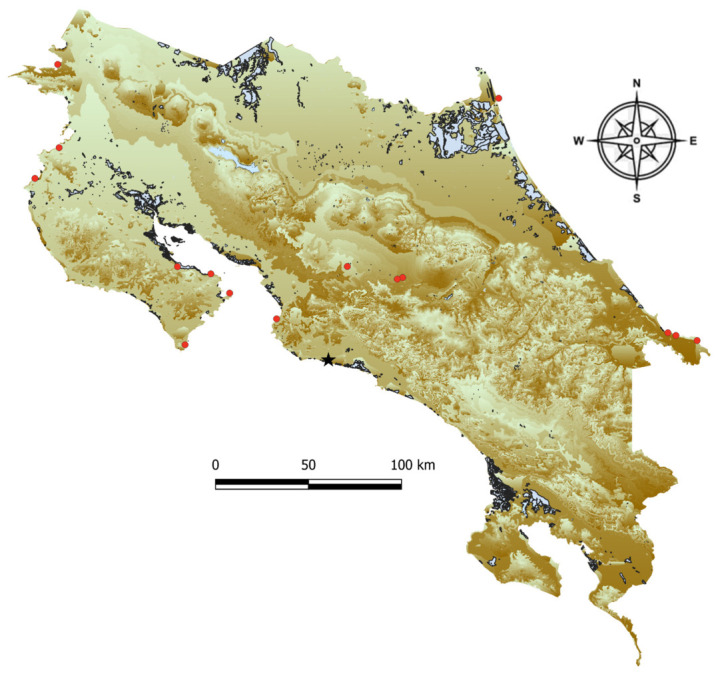
The location of the backyard chicken case detected on the Pacific coast (black star). The remaining red dots indicate the locations where AIV-positive wild birds were collected. The areas highlighted in blue represent wetland distribution; elevation is depicted in shades of green and brown. Green areas indicate elevations below 1000 m above sea level, and increasing brown intensity corresponds to elevations ranging from 1000 m up to the highest mountain peak at 3800 m above sea level.

During surveillance of backyard poultry near wetlands, officials from the National Veterinary Service visited a backyard poultry farm on 24 January 2023. The property was in Parrita County, Puntarenas Province, on the Pacific coast (Figure 1). During the visit, the owner reported that approximately 50 birds of different species, including ducks, chickens, quails, geese, and turkeys, had died during the previous week, representing a moderate mortality rate (41%). At the time of inspection, most birds appeared clinically normal; however, some exhibited respiratory distress and reduced appetite. Serum samples were collected from three chickens and one duck for laboratory analysis.

Based on the serological test results, a second visit was conducted on 27 January. During this visit, three SENASA officials wearing personal protective equipment (PPE), including Tyvek^®^ coveralls Wilmington, DE, USA, latex gloves, face masks, and boots, depopulated approximately 65 birds on the property using Euthanex (Invet, Cota, Bogotá, Colombia). Tissue samples and serum were collected from 30 birds. The premises were disinfected with Virkon (Zotal Laboratorios, Camas, Seville, Spain), and repopulation was permitted after two months. A pit was excavated using a municipal backhoe to bury the carcasses, contaminated equipment, and other waste materials.

### 2.1. Diagnostic Testing

#### 2.1.1. ELISA to Detect IgG AIV Antibodies

IDvet Screen^®^ Influenza A Antibody Competition Multi-species ELISA (ID Grabels, Montpellier, France) was used to detect IgG antibodies by following the manufacturer’s instructions. Any positive or suspicious results were confirmed by agar gel immunodiffusion AGID, using both positive and negative controls.

#### 2.1.2. qRT-PCR Assays for Detection and Subtyping

Quantitative reverse transcriptase–polymerase chain reaction (qRT-PCR) to detect influenza type A was performed in the LSE-LANASEVE Lab, using primers and probe sequences previously described by Spackman et al. [8]. RNA was extracted by using either the Blood and Tissue Kit (Qiagen, Valencia, CA, USA) or magnetic beads (Applied Biosystems, Thermo Fisher Scientific, Waltham, MA, USA). Reverse transcription and amplification were carried out by using the OneStep RT-PCR Kit (Qiagen, Valencia, CA, USA) in a 15 µL reaction containing 1× OneStep buffer mix, 1.25 mM MgCl_2_, 0.32 mM dNTPs, 1.2 µM of a primer mix (M25F, 124R [8], and 124R SIV [9], 0.12 µM of the M64 FAM-labeled probe, 1× ROX dye, 1× OneStep RT mix, and 8 µL of extracted RNA.

Lungs, cecal tonsil, and heart samples were processed at Inciensa Laboratory within a One Health framework, using the Centers for Disease Control and Prevention (CDC) qRT-PCR subtyping primer and probe kits to detect Influenza Virus A/H5a; AIV-H5b (Asian lineage) for research use only (RUO) (Catalog No. FluRUO-13) FR-1712 and Influenza Virus A/H7 (Eurasian lineage) (RUO) (RUO) FR-1258 were used according to the instructions provided by the CDC. The enzyme mix and reaction buffer of Applied Biosystems™ AgPath-ID™ One-Step RT-PCR kit (catalog No. 4387391) was used. A 25 µL reaction was used, consisting of 5.5 µL nuclease-free water, 0.5 µL forward primer, 0.5 µL reverse primer, 0.5 µL probe, 0.5 µL RT-PCR enzyme mix, 12.5 µL 2X RT-PCR buffer, and 5 µL RNA. The positive controls included the CDC Influenza A/H5N1 (Asian Lineage) Real-Time RT-PCR Positive Control with Human Cell Material (RUO) (Catalog No. VA2715) FR-176 and the CDC Influenza A/H7 (Eurasian Lineage) Positive Control (EuH7PC) (RUO) (Catalog No. KK0818) FR-1257. All CDC kits and reagents were obtained through the International Reagent Resource, Influenza Division of the World Health Organization (WHO) Collaborating Center for Surveillance, Epidemiology and Control of Influenza at the Centers for Disease Control and Prevention in Atlanta, GA, USA.

### 2.2. Genome Sequencing

The CDC’s multifragment RT–PCR protocol was used for the genomic amplification of the influenza A virus. Primers Uni12/Inf-1 (5′GGG GGG AGC AAA AGC AGG-3), Uni12/Infl-3 (5′GGG GGG AGC GAA AGC AGG-3), and Uni13/Infl-1 (5′CGG GTT ATT AGT AGA AAC AAG G-3), as well as SuperScript™ III One-Step RT–PCR System with Platinum™ Taq High Fidelity DNA Polymerase (Invitrogen-Thermo Fisher Scientific, Waltham, MA USA) were used in a conventional RT–PCR assay [10]. Briefly, 12.5 µL of 2X Buffer was mixed with 1 µL of a primer pool (20 µL of Primer Uni12/Infl-1 10 µM, 30 µL of Primer Uni13/Infl-1 10 µM, and 50 µL of Primer Uni13/Infl-1 10 µM), 0.5 of the 50X Enzyme Superscript III Polymerase, and 8 µL of DEPC water. Then, 3 µL of purified RNA was added to the mix. The following RT–PCR thermal cycling program was used: 1 cycle at 42 °C for 50 min, 50 °C for 10 min, and 94 °C for 2 min; 4 cycles at 94 °C for 30 s, 43 °C for 30 s, and 68 °C for 3 min and 50 s; and a final stage of 30 cycles at 94 °C for 30 s, 57 °C for 30 s, and 68 °C for 3 min and 50 s. The time of the last extension step was increased by 10 s in each cycle, with a final extension at 68 °C for 10 min. The amplified product was analyzed by using a QIAxcel Connect capillary electrophoresis instrument (QIAGEN; Germantown, MD, USA).

The DNA library was prepared by using the Illumina DNA Prep kit, following the manufacturer’s instructions (Illumina DNA Prep Reference Guide Document 25416 v10, San Diego, CA, USA).

### 2.3. Virus Isolation and Confirmatory Next-Generation Sequencing Analysis

A duplicate sample consisting of a pooled lung and trachea homogenate was sent to the WHO Collaborating Center (WHOCC) at St. Jude Children’s Research Hospital (SJCRH) for virus isolation and next-generation sequencing (NGS) analysis. Briefly, the pooled lung and trachea homogenate sample was transferred to the Animal Biosafety Level 3+ lab at SJCRH where it was propagated in 10-day-old embryonated chicken eggs and incubated at 35 °C. After incubation, the eggs were candled at 24 and 40 h post-inoculation. Once embryo death was observed, the eggs were chilled at 4 °C overnight. The allantoic fluid was harvested to test for influenza virus by hemagglutination assay (HA) using 0.5% chicken erythrocytes according to WHO protocol. The virus isolate was subsequently characterized via nucleic acid extraction and full genome sequencing. NGS analysis was performed using CLC Genomics Workbench 23.02 using a workflow that merged overlapping pairs, trimmed the reads, and mapped the reads to reference sequences to HPAI. A consensus of all segments was created and exported to a fasta file that could be blasted for comparison to other HPAI using NCBI. Nextclade was used for clade determination, and Genoflu for genotype.

### 2.4. Phylogenetic Analysis

Phylogenetic analyses incorporated 12 sequences from the 2023 H5N1 outbreak in Costa Rica, including two complete genomes of strain D0374-23 corresponding to pre-embryonic chicken egg isolation (BECEI) and post-embryonic chicken egg isolation (AECEI) stages. This dataset was supplemented with 78 sequences retrieved from GenBank [11] and 6 from the GISAID database [12]. To select reference sequences, the Costa Rican outbreak sequences were queried against the GenBank and GISAID databases using BLAST v2.17.0, and the seven most similar sequences to each Costa Rican sequence were downloaded for subsequent phylogenetic analysis. The EPI_ISL_1254_goose_CHN_1996 was selected as the outgroup for the phylogenetic analyses. The final set of 96 sequences was concatenated utilizing BioEdit v7.7.1 [13]. The whole-genome sequences were aligned by using the online software Clustal Omega v1.2.4 [14]. A phylogenetic tree was then constructed in MEGA X [15] by using the GTR+I model as the best-fit nucleotide substitution model, as determined by the Bayesian Information Criterion. The tree was inferred using the maximum likelihood method with 1000 bootstrap replicates in MEGA X [15]. An additional phylogenetic tree based on the HA gene was constructed using the KHY+G substitution model and the maximum likelihood method with 1000 bootstrap replicates, including the 96 sequences described above. For this analysis, five additional sequences from Costa Rica were included: three collected from the Pacific coast of Costa Rica, and two, from the Atlantic coast. The software Sequence Demarcation Tool (SDT v1.3) [16] was used to determine the pairwise identity scores of all sequences included in the study.

## 3. Results

### 3.1. Backyard Chickens

Of the four serum samples collected on 24 January during the initial farm visit, the three chicken (*Gallus gallus domesticus*) samples tested negative for influenza A, whereas the duck (*Anas platyrhynchos domesticus*) sample tested positive by ELISA and AGID at LSE-LANASEVE. A follow-up visit was conducted on 27 January. During this visit, a total of 30 chickens were sampled, and three pools of 10 oropharyngeal swabs were collected. In addition, lungs, cecal tonsils, livers, and hearts from the deceased birds were submitted to the laboratory, along with four additional serum samples. The remaining birds on the farm were subsequently culled.

Of the four chicken serum samples collected during the second visit, one tested negative for influenza A, while the remaining three were positive by both ELISA and AGID. Table 1 presents the results of the qRT-PCR assays performed at LSE-LANASEVE using the United States Department of Agriculture (USDA) qRT-PCR protocol for the detection of avian influenza virus type A. The USDA qRT-PCR assay detected influenza A in pooled samples from the heart, liver, lung, and cecal tonsils, with Ct values ranging from 31.7 in the lung to 36.0 in the liver, whereas all swab and trachea samples tested negative. Three pooled samples from the lung, trachea, and cecal tonsils were subsequently sent to INCIENSA for analysis using the Centers for Disease Control and Prevention (CDC) qRT-PCR protocol. According to the AIV type A and H5 qRT-PCR results shown in Table 1, the lung sample had the highest viral load.

The duck serum collected on 24 January and three chicken sera collected on January 27, with a duplicate pooled sample of cecal tonsils, lungs, and trachea, were sent to the National Veterinary Services Laboratories (NVSL) in Ames, IA, USA. The sera were used for subtype identification by hemagglutination inhibition (HI) assay, and the pooled samples were used for qRT-PCR analysis.

The results obtained from the NVSL indicated that the duck serum was positive for low pathogenic avian influenza virus (LPAIV) H5, with a titer of 1:64. The three chicken sera collected during the second visit also reacted to North American H5 LPAIV antigens: chicken serum 1 had a titer of 1:64; chicken serum 3 showed reactivity at 1:32; and chicken serum 4 was positive at 1:512, all corresponding to the H5 subtype. For those samples with limited volume, testing with North American and Eurasian H5 antigens was prioritized. Strong reactivity (≥1:32) to North American H5 antigens (H5N8 and/or H5N9 LPAIV) was observed in all four serum specimens. Only chicken serum 4 had sufficient volume for endpoint titration, yielding a titer greater than 1:8000 against the North American H5 LPAIV antigen. Weak cross-reactivity to other antigens was also observed, which is not unexpected in such cases.

The pooled cecal tonsil, trachea, and lung samples were negative by virus isolation, and only the lung was positive by qRT-PCR for influenza A (Ct 37.1) and for N1 (Ct 37.2). The NVSL result indicated that weak detection of influenza A could not be ruled out for one of three specimens; however, H5-clade-2.3.4.4-specific assays were negative, no virus was isolated, and serologic testing identified strong reactivity to North American H5 LPAI.

### 3.2. Viral Genome Sequencing

The extracted lung sample sent to Inciensa for NGS generated data for five of the eight genomic segments; the sequence was identified as D0374-BECEI-23. However, the average sequencing depth per segment was relatively low: HA (67.8x), NA (4.5x), MP (11.3x), NS (21x), NP (4.8x), PB1 (-), PB2 (-), and PA (-). Repeat sequencing of a new extracted sample of lung and cecal tonsil gave the following results: HA (225x), NA (6.7x), MP (211x), NS (236x), NP (39), PB1 (45.6x), PB2 (9.3x), and PA (17.2x). The HA amino acid sequence contained multiple basic residues at the HA cleavage site (HACS) between the HA1 and HA2 domains (REKRRKR↓G), confirming that this strain corresponds to a highly pathogenic avian H5 influenza virus. The HA amino acid sequence is shown in the Supplementary Materials (Figure S1).

A duplicate lung tissue sample was sent to the WHOCC at SJCRH for confirmation of virus sequence and virus isolation. Viral RNA was extracted from the virus containing allantoic fluid by using an RNAeasy Mini Kint (Qiagen). Amplification of all eight virus gene segments was achieved using the primers and conditions described previously [10]. Sequencing libraries were generated from purified PCR products (purified using Qiagen Qiaquick PCR purification kit) by using a Nextera XT DNA Library Prep Kit (Illumina) according to the manufacturer’s protocol and sequenced using a Novaseq pipeline (Illumina). NGS analysis was performed using CLC Genomics Workbench 23.02 using a workflow that merged overlapping pairs, trimmed the reads, and mapped the reads to reference sequences. A consensus of all segments was created and exported to a fasta file. The full sequence of all eight genomic segments was recovered with high coverage: HA (501,920x), NA (336,201x), MP (790,703x), NS (920,551x), NP (426,221x), PB1 (539,259x), PB2 (334,997x), and PA (323,926x). The sequence D0374AECEI-23 corresponds to the isolate generated through this process.

### 3.3. Phylogenetic Analyses

**Figure 2:**
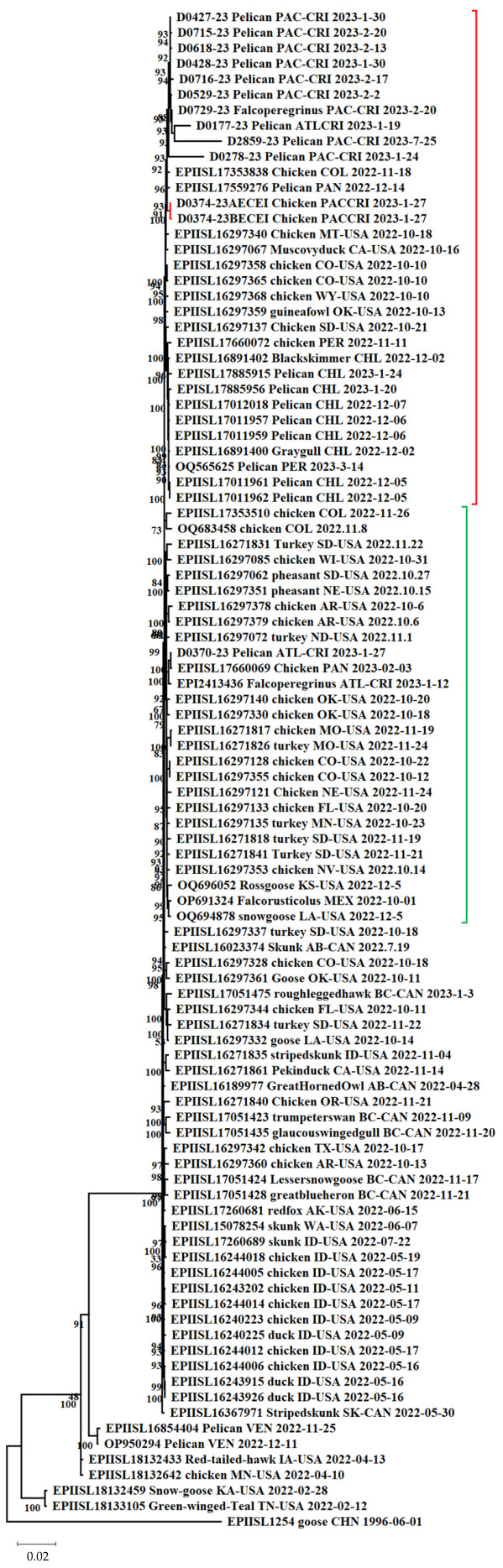
The maximum likelihood phylogenetic tree constructed from the concatenated whole-genome sequences (13,136 nucleotides) of 96 avian influenza viruses. The green bracket highlights the sequences obtained from wild birds on the Atlantic coast of Costa Rica, whereas the red bracket indicates the sequences obtained from wild birds and the chicken on the Pacific coast of Costa Rica. The distance in the scale bar represents the among of nucleotide substitutions per site, in this case 2 substitutions per 100 nucleotides.

Phylogenetic analyses were conducted on the concatenated sequences generated in this study and those in public sequence databases (Figure 2). The Costa Rican sequences were distributed into two clusters. One cluster (highlighted with a red bracket in Figure 2) comprised 34 sequences including 10 sequences collected from the Pacific coast of Costa Rica, among them D0374BECEI-23 and D0374-23AECEI (before and after the embryonic chicken egg isolation process; branch highlighted in orange). These two sequences grouped together with a bootstrap value of 72 and shared a common ancestor with the remaining Costa Rican sequences.

The other cluster (highlighted with a green bracket in Figure 2) included 27 sequences. Within this cluster, two sequences from Costa Rica, isolated from wild birds on the Atlantic coast, were grouped with a sequence isolated from chickens in Panama. The Atlantic cluster also included sequences from Mexico (n = 1), Colombia (n = 2), and 13 USA sequences. In addition to these two main clusters, the phylogenetic tree was arbitrarily divided into five additional groups, labeled 4 to 8. The sequence EPI_ISL_1254, isolated from a goose in China in 1996, was used as the outgroup.

Pairwise identity scores generated by the Sequence Demarcation Tool (SDT v1.3) revealed an average nucleotide identity (ANI) of 99.964% between the D0374-23BECEI and D0374-23AECEI sequences, representing a minimal divergence of 0.036% (five nucleotide differences across an 11,577-nucleotide [nt] overlapping region). Out of the 13,136 nt comprising the complete D0374-23AECEI genome, the sequence recovered directly from infected tissue (D0374-23BECEI) lacked 1368 nt in the PB1 gene and 191 nt in the HA gene, accounting for the reduced consensus length. Furthermore, nucleotide divergence between the Costa Rican chicken sequences and the outgroup subtype H5N1 clade 0 EPI_ISL_1254_goose_CHN_1996 was 11.82%. In contrast, these sequences exhibited a minor divergence of 0.63% (83 nt) against the CRATL cluster, and 0.57% (75 nt) against the remaining sequences within the CRPAC cluster.

**Figure 3:**
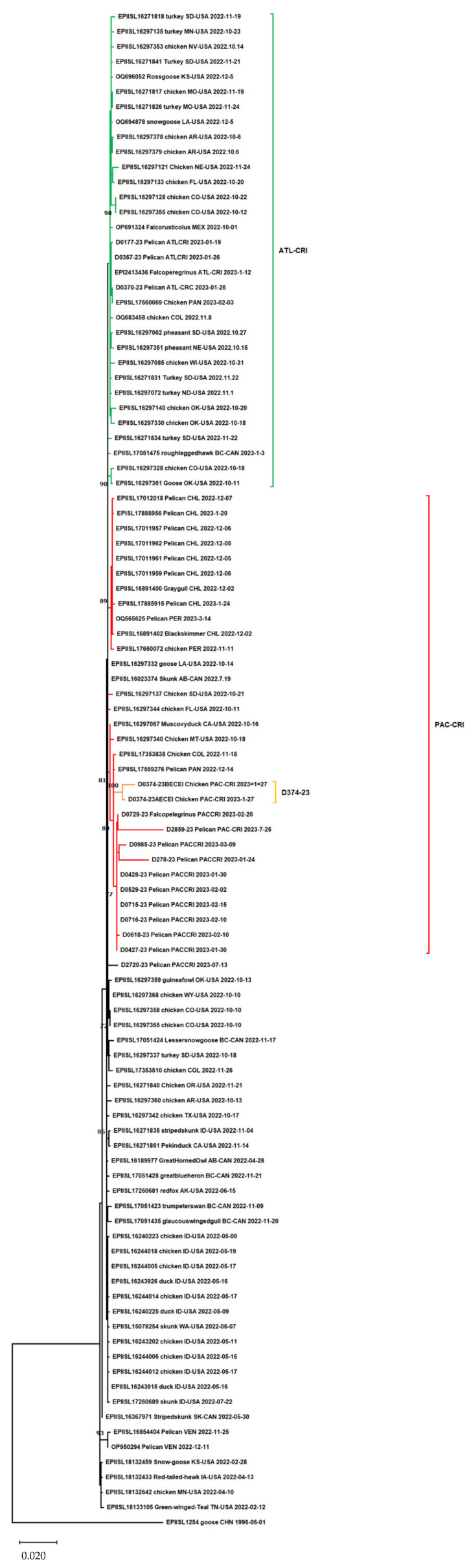
The phylogenetic tree of the HA gene. The green bracket highlights the sequences from Costa Rica collected on the Atlantic coast; the red bracket indicates sequences isolated from the Pacific coast. The orange branch represents sequences obtained from chickens in Costa Rica. The bar scale is 0.2 substitutions per 100 nucleotides.

Figure 3 illustrates the phylogenetic distribution of 16 HA-AIV sequences isolated in Costa Rica. Among these, ten sequences derived from wild birds and two from the same backyard chickens clustered together with sequences from Colombia, Panama, Peru, Chile, USA and Canada. As in the concatenated sequences the D374-23 HA gene sequences cluster together with a boostrap value of 100.

Conversely, four other wild bird Costa Rican sequences clustered with a sequence from Colombia, Panama, Mexico, Canada and several from the USA. In both the whole-genome and HA phylogenies, the backyard chicken sequences were distant from the remaining Costa Rican cohorts. Notably, within the HA tree, these specific chicken sequences exhibited a closer phylogenetic relationship to a Colombian isolate than to those collected domestically in Costa Rica.

## 4. Discussion

In the context of the global expansion of H5N1 clade 2.3.4.4b viruses, the case detected in Parrita, Costa Rica, provides relevant insight into avian influenza virus dynamics at the interface between domestic poultry and wild birds. Since its emergence from the Gs/GD lineage in Asia [17], H5N1 has become established in domestic and wild bird populations, with migratory species contributing to its geographic spread [18]. The southward movement of clade 2.3.4.4b viruses into Central and South America during 2022–2023 has been associated with multiple outbreaks in both wild and domestic birds across the region [7,18]. This expansion is part of the global dissemination of the Guangdong/1/1996 (Gs/GD) lineage, which was initially driven by spillover transmission from poultry to wild birds and subsequently sustained through continued circulation in wild bird populations [19,20,21]. A hallmark of Gs/GD-lineage HPAIVs is the presence of multibasic amino acids in the HACS. In our isolate, the HACS motif consisted of eight amino acids (REKRRKRG), six of which were basic residues. This multibasic motif is recognized and cleaved by the ubiquitous proprotein convertase furin, enabling activation of the HA0 precursor and facilitating systemic viral replication in a wide range of tissues and cell types, a key molecular determinant of high pathogenicity [20].

The distribution of the Atlantic and Pacific sequences into distinct clusters is particularly interesting given the short distance between the two coasts of Costa Rica. This pattern suggests that these strains have different origins and likely reflect two migratory bird flyways: the Pacific Flyway along the Pacific coast of the Americas and the Central Flyway, which passes through the Caribbean (Atlantic) coast of Costa Rica. The placement of the chicken sequence within the Pacific cluster, while remaining genetically distinct from the other Costa Rican sequences, is also noteworthy and may indicate a different source of introduction. Genomic analyses confirmed the presence of HPAIV H5N1 clade 2.3.4.4b in backyard poultry from Parrita, based on molecular features consistent with high pathogenicity. However, the clinical presentation, serological findings, and virological results observed in this case do not fully align with those typically described in naïve poultry populations affected by this clade.

The detection of antibodies against H5 LPAIV in both a domestic duck and backyard chickens represents an unusual finding in the Costa Rican context, where previous serological evidence of AIV circulation has not been documented [22]. Waterfowl (Anseriformes) are recognized reservoirs of LPAIV and can shed the virus in high concentrations, particularly through feces [19,23]. Exposure through environmental contamination, including water sources, has been described as an efficient transmission route between wild and domestic birds [24,25]. In this situation, prior exposure of the flock to low pathogenic avian influenza virus (LPAIV) cannot be ruled out, nor can the possibility that the antigen used in the hemagglutination test cross-reacted with clade 2.3.4.4b viruses.

The temporal pattern of antibody detection, with initial seropositivity limited to the duck and subsequent detection in chickens, may be consistent with differences in exposure or infection dynamics among species within the flock; several experiment and clinical studies considered ducks as the natural carriers of the AI virus, as ducks infected with avian influenza viruses did not develop clinical diseases, and may play an efficient role in the maintenance and dissemination of LPAIV, but also of H5-HPAIV, typically associated with lower mortalities as compared to other duck species [26,27]. Additionally, the observed mortality rate (~41%) appears to be lower than that reported in some outbreaks involving HPAIV clade 2.3.4.4b in immunologically naïve populations. In an experimental study conducted in 1-week-old ducks, all birds died following a single HPAIV inoculation. However, prior infection with H6N6 and H9N2 LPAIVs increased the survival rate of domestic ducks after subsequent H5N6 HPAIV infection. Nevertheless, all surviving ducks with prior LPAIV infection seroconverted only to the homologous strain, and the induced neutralizing antibodies against the LPAIVs were not responsible for protection against heterologous HPAIV infection [28].

In another experimental study, three groups of captive mallards (*Anas platyrhynchos*) were evaluated: (i) fully susceptible birds, (ii) birds pre-exposed to low pathogenic avian influenza virus (LPAIV) subtype H5N1, and (iii) birds pre-exposed to LPAIV subtype H3N8. In the susceptible group, approximately 60% mortality was observed, along with neurological signs, abundant viral shedding, and transmission to contact ducks, which also became sick and died.

Mallards pre-exposed to LPAIV H5N1 and subsequently challenged with HPAIV H5N8 remained asymptomatic and showed a significant reduction in viral RNA shedding, although shedding was still sufficient to infect contact ducks, which, however, did not develop disease. Similarly, mallards previously inoculated with LPAIV H3N8 remained clinically healthy after challenge with HPAIV H5N8, but high levels of viral RNA were detected in swabs and organs. Antibody levels against homologous antigens increased following inoculation with the LPAIV strains [29]. However, given the limited sample size and observational nature of our data, caution is warranted when interpreting these findings.

Virological results further contribute to this complexity. High Ct values (31.7–37.2) and low viral loads reported by SENASA and USDA laboratories [30] may be indicative of reduced viral RNA levels in sampled tissues. Similarly, the inability to isolate the virus and incomplete amplification of the HA gene could reflect low viral titers or sample degradation. These findings are consistent with scenarios described in previous studies in which viral detection is limited despite confirmed infection [29].

Experimental studies have shown that prior exposure to LPAIV may influence the outcome of subsequent HPAIV infection in waterfowl, including by increasing survival and reducing viral shedding [28]. Although such mechanisms could be relevant in the present case, the available data do not allow for direct assessment of immune-mediated protection or causality.

The possibility of laboratory cross-contamination was considered; however, this appears to be unlikely given that the obtained sequences did not match other samples processed during the same period, and independent sequencing with higher coverage confirmed the presence of HPAIV in the analyzed tissue. Moreover, identity score analysis demonstrated that the sequences obtained from backyard chickens are distinct from the other Costa Rican sequences, despite the overall similarity observed among them. Furthermore, the high-coverage sequences obtained by St. Jude Children’s Research Hospital corroborated the initial findings from Inciensa, confirming that the HPAIV strain was indeed present in the tissue. Despite the low deep coverage of the D0374-23BECEI sequence obtained by Inciensa directly from the infected tissue, only five nucleotide differ from the sequences obtained by the WHO Collaborating Center (WHOCC) at St. Jude Children’s Research Hospital.

Taken together, these findings suggest that multiple epidemiological and biological factors may have contributed to the observed patterns in this outbreak. The coexistence of serological evidence of LPAIV exposure and molecular confirmation of HPAIV highlights the complexity of AIV dynamics in backyard production systems. Additionally, the potential for H5 and H7 LPAIV strains to evolve into highly pathogenic forms has been well documented, as in the H5N2 outbreak in Mexico during 1994–1995. Furthermore, AIV strains have been documented to spill over into neighboring Central American countries, including Guatemala and El Salvador [31], reinforcing the need for wild bird and backyard bird surveillance in the country.

## 5. Conclusions

This case underscores the importance of continued surveillance of both less and highly pathogenic AIV strains in domestic and wild bird populations. Our findings suggest that previous exposure to low pathogenic avian influenza viruses (LPAIVs) may have mitigated the clinical outcome of the subsequent H5N1 clade 2.3.4.4b infection in this backyard flock. The presence of LPAIV antibodies, the moderate mortality, the low viral loads detected in the tissues, and the unequivocal confirmation of HPAIV by whole genome sequencing support this hypothesis. These results highlight the potential role of pre-existing immunity in modifying the severity of HPAIV outbreaks and emphasize the importance of integrated serological and molecular surveillance of avian influenza viruses in domestic and wild birds.

## Figures and Tables

**Table 1:** qRT-PCR assay detection of influenza virus in the backyard bird samples.

		LSE-LANASEVE qRT-PCR USDA		Inciensa CDC qRT-PCR		
	**Sample Source**	**AIV-A**		**Inf A**	**AIV-H5a**	**AIV-H5b**
**Species**	**(Pooled)**			**Ct**	**Ct**	**Ct**
Chicken	Trachea	Not detected		32.18	36.76	36.74
Chicken	Lungs	Detected	31.77	24.74	25.95	27.42
Chicken	Cecal tonsil	Detected	32.46	25.83	27.39	28.37
Chicken	Heart	Detected	34.23	ND	ND	ND
Chicken	Liver	Suspicious	36.03	ND	ND	ND
Chicken	Swab	Not detected				

AIV-A: this PCR detected influenza virus type A. ND, not done; Ct, cycle threshold. Inf A corresponds to the CDC qRT-PCR to detect avian influenza type; AIV-H5a and AIV-H5b are the dual-target H5 assay designed to reduce the likelihood of false-negative results caused by genetic variation in circulating H5 viruses.

## Data Availability

Data Availability under request if applicable.

## Supplementary Materials

The following supporting information can be downloaded at https://www.mdpi.com/article/10.3390/v18070799/s1, Figure S1: D0374-23 HA sequences obtained by performing next-generation sequencing.
