# Supplementary material for: Detection of H5N1 HPAIV Clade 2.3.4.4b Avian Influenza Virus in Backyard Chickens in Costa Rica
Source: Viruses. 2026 Jul 20;18(7):799. doi: 10.3390/v18070799 (PMC13431611; doi:10.3390/v18070799)

Supplementary Material

Figure S1. shows the D0374-23 HA sequences obtained by performing next-generation sequencing; the red line highlights the cleavage site between the HA1 and HA2 domains.

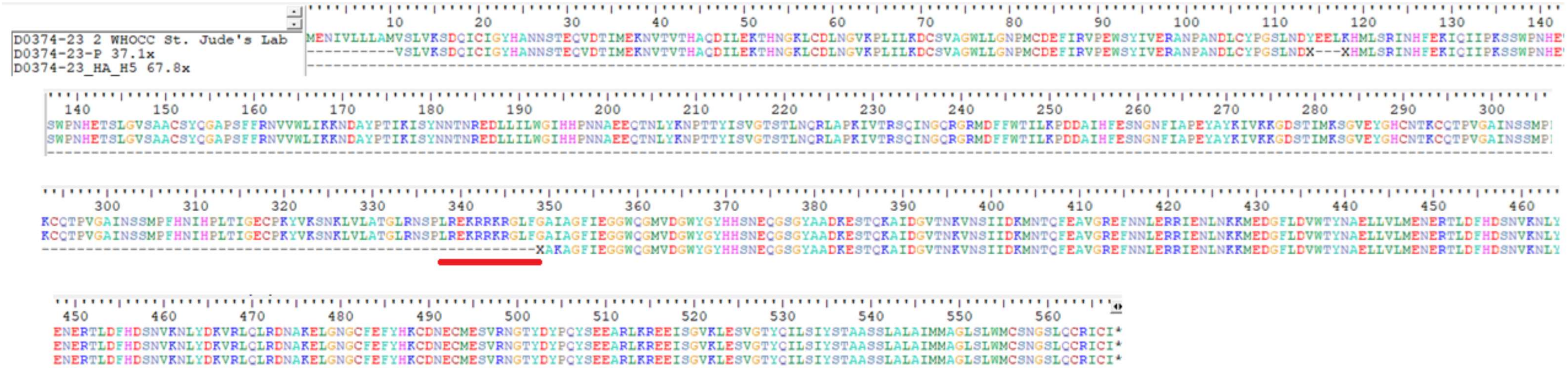

Supplement: Supplementary file 1 [file viruses-18-00799-s001.zip › viruses-4373593-supplementary.pdf]
